# Complete Genome Sequences of Six Lactobacilli Isolated from American Quarter Horses

**DOI:** 10.1128/MRA.00997-20

**Published:** 2020-11-19

**Authors:** Rachael I. Meinders, Mary Mendoza, Allison N. Dickey, Elizabeth H. Scholl, Hosni M. Hassan

**Affiliations:** a Prestage Department of Poultry Science, North Carolina State University, Raleigh, North Carolina, USA; b Bioinformatics Research Center, North Carolina State University, Raleigh, North Carolina, USA; University of Maryland School of Medicine

## Abstract

We report the complete circular genome sequences of six *Lactobacillus* strains and their plasmids, if any, from the fecal material of quarter horses at different ages.

## ANNOUNCEMENT

Horses are nonruminant herbivores, and fecal samples have been shown to represent changes in the midgut due to changes in diet, age, and health (1, 2). Here, fecal samples from three healthy horses were used to isolate six *Lactobacillus* strains. Isolates 1A, 1B, and 1D were from a 7.5-month-old female weanling fed a grain/grass diet; 2D was from a 6-month-old female foal (1 day postweaning) fed milk before switching to a grain/grass diet; and 3B and 3DG were from a >10-year-old mature male gelding fed a grass/hay diet.

Fresh fecal samples (less than 1 h old) were collected from pile centers, transferred to the laboratory on ice and processed (within 4 to 5 h). Samples were diluted to 100 mg/ml in MRS medium, inoculated 1:10 into MRS with and MRS without 1% galacto-oligosaccharides and cultured anaerobically at 37°C for 24 h (3, 4). Serial dilutions were plated onto solid MRS and incubated at 37°C for 48 h. From each sample, 4 to 6 colonies were selected based on colony morphology differences and biochemical tests. Gram-positive, catalase-negative, non-spore formers and nonmotile colonies were selected, grown in MRS, and stored at −80°C in MRS/25% glycerol. Frozen cells were cultured anaerobically in MRS for 18 h. The steps of DNA extraction through sequencing have been described previously (5).

The assemblies were generated using HGAP4 (SMRT Link v5.1.0) (6). Preliminary assemblies showed high coverage (>2,000×), and the reads were subsampled as described in reference 5 (with sampling fractions listed in Table 1) before generating the assemblies. Circlator v1.5.5 (assembler, Canu) (7) was used for circularization, and the assemblies were polished with the subsampled reads using Arrow (GenomicConsensus package v2.2.2 in SMRT Link). The 1B assembly included a 30,259-bp contig whose sequence closely matched segments in the chromosome between base pairs ∼883803 and 927416, and this contig is not included in the final assembly. The 1B plasmid has a region between base pairs ∼3384 and 6688 that had higher read coverage and single nucleotide polymorphisms (SNPs), and its annotation includes a tyrosine-type recombinase/integrase, an element found twice in the chromosome between base pairs ∼883803 and 927416.

**TABLE 1:** Subread and assembly summary^*a*^

Isolate	SRA accession no.	No. of subreads	Subread *N*_50_ (bp)	Sampling fraction	GenBank accession no.	Assembly genome length (bp)	Mean coverage (×)	Chromosome		Plasmid		No. of genes	Assigned *Lactobacillus* species	ANI reference genome
								Size (bp)	GC content (%)	Size (bp)	GC content (%)			
1A	SRR10752814	1,165,346	10,262	0.057	CP047418	1,900,000	227	1,802,611	42.6	ND	ND	1,838	*L. saerimneri*	*L. saerimneri* 30a
1B	SRR10752813	1,149,017	11,200	0.072	CP047416	2,400,000	220	2,243,550	38.9	29,164	40.1	2,284	*L. reuteri*	*L. reuteri* DSM 20016
					CP047417									
1D	SRR10752812	2,559,486	5,128	0.071	CP047415	2,200,000	219	2,349,358	36.9	ND	ND	2,383	*L. crispatus*	*L. crispatus* ST1
2D	SRR10752811	1,287,277	9,534	0.063	CP047412	1,850,000	200	1,700,858	33.2	248,523 (P1)	32.3 (P1)	2,008	*L. salivarius*	*L. salivarius* UCC118
					CP047413					33,778 (P2)	39.3 (P2)			
					CP047414									
3B	SRR10752810	1,183,161	7,819	0.097	CP047410	1,900,000	189	2,150,064	42.6	37,548	37.0	2,165	*Lactobacillus* sp.	ND
					CP047411									
3DG	SRR10752809	1,235,045	8,840	0.074	CP047409	2,000,000	203	1,995,616	34.4	ND	ND	1,929	*L. johnsonii*	*L. johnsonii* strain Byun-jo-01

a ND, no data; P1, plasmid 1; P2, plasmid 2.

The six isolate chromosomes and the 2D plasmids were circularized with Circlator. Read mapping to the 1B plasmid indicated that it was circular, and no circularization steps were performed. For 3B, after polishing the circularized chromosome and the plasmid as it was generated in the assembly, the polished plasmid ends had 8,627 bases of overlap. The overlapping bases were removed from one end, and the assembly was repolished.

NCBI PGAP v4.10 was used for annotation (8, 9). Except for 3B, the assemblies and the reference genomes of their assigned species had >95% average nucleotide identity (ANI). The ANI was calculated using CompareSketch from BBMap v38.82 (10).

### Data availability.

The sequencing reads and the assemblies are available under the accession numbers shown in Table 1.
